# *Staphylococcus aureus* Colonization: Modulation of Host Immune Response and Impact on Human Vaccine Design

**DOI:** 10.3389/fimmu.2013.00507

**Published:** 2014-01-08

**Authors:** Aisling F. Brown, John M. Leech, Thomas R. Rogers, Rachel M. McLoughlin

**Affiliations:** ^1^Host-Pathogen Interactions Group, School of Biochemistry and Immunology, Trinity Biomedical Sciences Institute, Dublin, Ireland; ^2^Sir Patrick Dun Laboratory, Department of Clinical Microbiology, Trinity College Dublin, St James’s Hospital, Dublin, Ireland

**Keywords:** *Staphylococcus aureus*, colonization, host response, immunomodulation, T cells, microbiota, vaccine

## Abstract

In apparent contrast to its invasive potential *Staphylococcus aureus* colonizes the anterior nares of 20–80% of the human population. The relationship between host and microbe appears particularly individualized and colonization status seems somehow predetermined. After decolonization, persistent carriers often become re-colonized with their prior *S. aureus* strain, whereas non-carriers resist experimental colonization. Efforts to identify factors facilitating colonization have thus far largely focused on the microorganism rather than on the human host. The host responds to *S. aureus* nasal colonization via local expression of anti-microbial peptides, lipids, and cytokines. Interplay with the co-existing microbiota also influences colonization and immune regulation. Transient or persistent *S. aureus* colonization induces specific systemic immune responses. Humoral responses are the most studied of these and little is known of cellular responses induced by colonization. Intriguingly, colonized patients who develop bacteremia may have a lower *S. aureus*-attributable mortality than their non-colonized counterparts. This could imply a staphylococcal-specific immune “priming” or immunomodulation occurring as a consequence of colonization and impacting on the outcome of infection. This has yet to be fully explored. An effective vaccine remains elusive. Anti-*S. aureus* vaccine strategies may need to drive both humoral and cellular immune responses to confer efficient protection. Understanding the influence of colonization on adaptive response is essential to intelligent vaccine design, and may determine the efficacy of vaccine-mediated immunity. Clinical trials should consider colonization status and the resulting impact of this on individual patient responses. We urgently need an increased appreciation of colonization and its modulation of host immunity.

## Introduction

*Staphylococcus aureus* can be a human commensal or a potentially lethal opportunistic pathogen. It is one of the leading causes of a variety of community-acquired and hospital-acquired bacterial infections. *S. aureus* is one of the most common causes of bacteremia, and carries a higher mortality than any other – 65–70% in the pre-antibiotic era, and currently 20–40% mortality at 30 days despite appropriate treatment (1, 2). It is also an important cause of other deep-seated infections including osteomyelitis, septic arthritis, endocarditis, device-related infections, and pneumonia. *S. aureus* is unusual for its propensity to cause primary bacteremia and serious infections among young, otherwise healthy people, as well as in those with risk factors (3). While invasive disease is by far the most acute and severe, the greatest burden of morbidity is due to skin and soft tissue infections (SSTIs), which are extremely common, often chronic, and frequently recurrent.

Invasive disease continues to occur despite improved adherence to infection prevention practices, and the organism has steadily evolved resistance to every licensed anti-staphylococcal agent to date. In this context, clinical need has driven research efforts toward strategies to develop an anti-*S. aureus* vaccine. Our lack of knowledge of what elements of the immune system are important in recovery from or prevention of human infection is staggering. This ignorance of what may constitute a protective immune response in humans makes designing vaccines even more challenging. Less than 10 candidates for passive or active immunization have progressed to clinical studies to date, and none have shown efficacy in preventing disease.

Intriguingly, despite its impressive armory and invasive opportunism, *S. aureus* replicates and evolves in a large proportion of the human population as a harmless colonizing organism and never causes disease. This review will explore interactions between colonizing *S. aureus* and the human immune system and describe the compelling impact colonization has on the risk and outcome of invasive *S. aureus* infection. Finally, we will consider the particular challenges of designing a vaccine against a colonizing organism and the importance of examining the potential priming effect of colonization in future clinical trials.

## Understanding Human *Staphylococcus aureus* Colonization

### Sites and patterns of *S. aureus* colonization

Humans are frequently exposed to *S. aureus* and it colonizes most of us, either for long or short periods at various stages throughout our lives. The primary *S. aureus* reservoir in humans is the anterior nares. Extra-nasal colonization sites include skin, throat, perineum, vagina, and gastrointestinal tract (4–6). Exclusively sampling nasal sites to determine whether a person is colonized at a single point in time will miss 50% of those colonized elsewhere (7). Nonetheless it appears that the nasal site is often the source of inoculation of other sites via hand transfer, and the greater the bacterial load in the nares, the higher the likelihood that other body sites are colonized and that the colonization is persistent (8–10). We will largely focus on the role of nasal carriage in this review as that is what has been most extensively investigated.

Nasal carriers may fall into two categories – persistent carriers and non-persistent carriers (11). Approximately 20% of individuals are persistently colonized with a relatively high bacterial load, and the remainder are either never colonized or only intermittently with low numbers of bacteria (12). Much of the existing literature examining the role of colonization is weakened by sampling participants at a single time point only, precluding differentiation between persistent and intermittent carriers. There is no standard definition of how many cultures should be taken and what fraction should be positive before determining carrier status, despite various proposals (9). This is unfortunate as it seems that the differences between persistent and non-persistent carriage patterns are critical in determining the risk of subsequent infection and may thus influence the nature of response to potential candidate vaccines (11).

### Transmission, dynamics, and natural history of colonization

A small minority (<5%) of neonates are colonized by *S. aureus* at birth, mainly if born by normal delivery in a vaginally colonized mother (6). In the first 2 weeks of life, colonization with mainly maternal strains rapidly occurs in half of infants, but this falls to adult rates by 6 months of age, coincident with the development of acquired immunity (13). One quarter of neonates are not colonized by *S. aureus* at all in the first 2 years of life, and what determines this resistance to acquisition is unknown.

Transmission of *S. aureus* occurs almost exclusively as a result of direct skin-to-skin contact, or contact with recently contaminated fomites (14, 15). Nonetheless, even proven contact with the organism does not necessarily result in subsequent colonization, and certain hosts remain non-carriers. In one study, only a minority of patients with prior culture-proven *S. aureus* skin infection remained colonized with the organism in the convalescent phase, and only 25% of their household contacts were colonized with the index infecting strain (7). Colonization rates among pre- and post-clinical medical students are not altered by increased exposure of the post-clinical group to *S. aureus* in healthcare settings (16). This suggests important host differences may confer resistance to colonization.

The relationship between each host and their colonizing strain is extremely personalized. The duration of colonization among nasal carriers has been measured from 70 days to 8 years. It seems longer among persistent carriers, although methods used to prove the isolate remains the same were suboptimal in comparison to contemporary techniques (11, 17–20). More recently, whole-genome sequencing has allowed deeper exploration of carried strains. It seems that nasal colonization results from a single founding organism that multiplies over time with the evolution of limited minor genetic variations (21). Persistent carriers may sequentially acquire a new strain that replaces their original colonizing organism (22). Experimental inoculation of established persistent and non-carriers with multiple strains resulted in most volunteers returning to their original “natural” carrier state, and sometimes even reverting to their original colonizing strain (11, 23).

### Risk factors for colonization

The prevalence of *S. aureus* in the anterior nares of a sample of healthy Europeans at a single time point was 21.6%, with slightly higher rates in men and younger adults (24). Accurate assessments of persistent carriage are more difficult to determine, but among relatively healthy adults chronic skin disease, recent skin infection, male sex, and being a non-smoker are associated with increased nasal colonization rates (25, 26).

Certain patient populations tend to have higher rates of colonization than healthy adults. Almost all (>90%) adult patients with atopic dermatitis (AD) are *S. aureus* nares and/or skin carriers (27). Granulomatosis with polyangiitis (GPA – formerly Wegener’s granulomatosis) patients also have higher rates of nasal *S. aureus* carriage (28). Other cohorts with recurrent skin breaches have higher carriage rates, including insulin-dependent diabetics, renal replacement therapy patients, and intravenous drug users, although the exact mechanisms are unclear (12). The rates among non-AD patients receiving injected allergen immunotherapy are not significantly higher than healthy controls, so perhaps repeated skin breaks alone are not sufficient to influence carriage (29). HIV-positive patients also appear to have more frequent nasal colonization, although there are many potential confounders which may explain this, including increased contact with healthcare, repeated anti-microbial exposures, tendency to develop skin disease, use of medical intravascular devices, and higher frequency of intravenous drug use. Nonetheless, even when corrected for degree of immunosuppression, viral load, and drug use, HIV remains an independent risk factor for *S. aureus* colonization for as yet undefined reasons (30, 31).

### The relationship between colonization and invasive disease

Nasal carriage of *S. aureus* is strongly associated with infection. Clinical studies consistently describe a significantly greater risk of bacteremia among carriers, quoting relative risks from 1.2 to 21.7 in cohorts with regular healthcare contact, especially in the presence of indwelling devices (12, 32–35). The majority (>80%) of *S. aureus* nosocomial bacteremias are caused by invasion of the endogenous colonizing strain (36, 37). Nasal carriage has also been shown to increase non-bacteremic *S. aureus* healthcare-associated infections, again largely with endogenous strains (38–41). While it seems logical that persistent carriers would have a greater risk of infection than those with transient carriage, this has rarely been formally tested. A single study of 52 peritoneal dialysis patients attempted to answer this question by measuring exit site infections and peritonitis (42). For both outcomes, the relative risk of clinically evident *S. aureus* infection was ninefold higher among persistent carriers as compared with non-persistent carriers. In the community, where the burden of disease is SSTIs, the colonizing strain is also the causative agent (43).

Colonization with methicillin-resistant *S. aureus* (MRSA) strains seems to confer a higher risk of subsequent invasion in hospitalized patients than methicillin-sensitive (MSSA) strains, although these patients may be inherently more complex with longer hospital stays and broader anti-microbial exposure (32, 44, 45). A study which matched patients for MRSA nasal carriage found that those with multiple hospitalizations or a central venous catheter *in situ* were more likely to develop *S. aureus* bacteremia, which may explain the aforementioned findings (35). In MSSA bacteremia the presence of a central venous catheter does not seem to make a significant difference, and colonization seems to be the much greater risk factor (37). Carriage of the North American community-acquired methicillin-resistant (CA-MRSA) strains may be associated with higher risk of SSTIs than MSSA carriage (46, 47). This is perhaps not surprising as these strains are often armed with several toxins ideal for tissue destruction (48). It does not appear to translate into a worse outcome in bacteremia, however (49). While the rise in nasal colonization with USA300 in North America is remarkable, the expansion of epidemic clones is not a new feature in the history of *S. aureus*, and mechanisms by which particular strains may be better colonizers have not been ascertained (50, 51). Clonal distributions in other parts of the world remain diverse (52, 53).

Despite their increased risk of infection relative to non-carriers, only a tiny minority of nasally colonized individuals actually suffer any adverse effects from their co-existence with *S. aureus*. Even with conservative estimates, the incidence of carriage is 1000 times greater than that of serious invasive infection (54). Despite its armory of virulence factors, the energy and time of *S. aureus* is overwhelmingly directed, not at causing invasive disease, but rather in spreading from host to host to establish colonization.

## Host Factors Determining *S. aureus* Colonization

**Figure 1:**
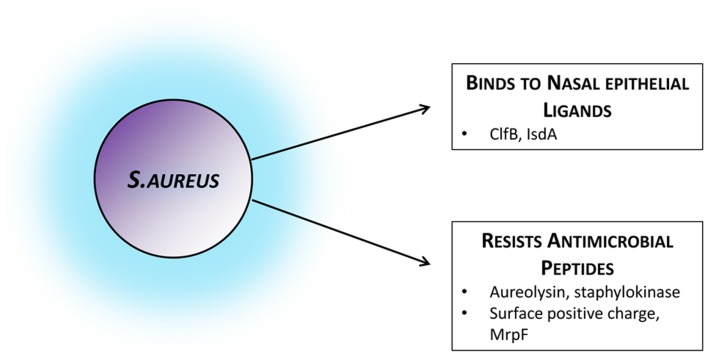
***Staphylococcus aureus* factors facilitating colonization**. Bacterial strategies and attributes known to facilitate colonization by mediating adhesion to the nasal epithelium or by actively evading host mechanisms of bacterial clearance. Those established in colonization settings and *in vivo* have been included, although several other immune-evasion mechanisms have been described in infection models or *in vitro*. ClfB, clumping factor B; IsdA, iron-regulated surface determinant A; MrpF, multiple resistance and pH regulation protein F.

Given the link between colonization and disease, strategies to prevent nasal colonization could be an appealing method of combatting *S. aureus* infection. In contrast to the multiple bacterial factors known to be involved in colonization (see Figure 1), there is far less known about host elements and their relative contribution.

### Anatomy and adherence

*Staphylococcus aureus* preferentially colonizes the vestibulum nasi. Autopsy studies have shown it residing within the squamous epithelial layer, associated keratin and mucous debris, and even within hair follicle shafts (55). Adherence of a laboratory *S. aureus* strain to the squamous cells of volunteers known to be naturally colonized is significantly greater than adherence to cells of non-carriers (56). Nasal secretions from human carriers also improve *in vitro* adherence, perhaps in part due to the presence of hemoglobin, which seems to inhibit *agr* expression (57).

During successful human colonization *S. aureus* preferentially expresses tissue-adherence and immune-evasion molecules and down-regulates virulence factors and toxins (58, 59). A number of microbial surface components recognizing adhesive matrix molecules (MSCRAMMs) have been shown to interact directly with the nasal epithelium. In particular clumping factor B (ClfB) and iron-regulated surface determinant A (IsdA) are factors thought to promote adhesion. Their host target ligands, however, are not as well-studied. ClfB adheres to cytokeratin K10 *in vitro*, and produces more successful experimental nasal colonization in humans (60). The key ClfB ligand *in vivo* is most likely loricrin. Loricrin is the most abundant protein in the keratinized epithelial layer of the nares. Binding of *S. aureus* to these human cells has recently been shown to be ClfB-dependent, and the absence of either loricrin or ClfB significantly impairs colonization in a murine model (61). This introduces the intriguing idea of blocking loricrin to inhibit colonization. Although born with skin abnormalities, due to multiple compensation mechanisms, loricrin deficiency in adult mice does not lead to significant phenotypic abnormality (62). Mutations in humans, however, are associated with significant diffuse skin disease (63). In the case of IsdA the human ligands involved are less clear and its effect on human colonization has not been convincingly demonstrated *in vivo* (64–66).

### Local host immune environment may determine colonization

The anti-microbial defense mechanisms of epithelial sites comprise a collection of host-defense lipids, peptides, and proteins produced by epithelial cells and immune cells recruited to the site (67). These have broad-spectrum anti-microbial activity and the ability to rapidly and directly kill organisms, and modulate the innate immune response (68). Some of these anti-microbial peptides (AMPs) have been shown to interact with *S. aureus*, but knowledge of their exact mechanisms of action and of their influence on nasal colonization is limited.

Nasal secretions from carriers contain higher concentrations of α-defensins (human neutrophil peptides 1–3) and human β-defensin 2, possibly as a consequence of the organism’s presence (69). These secretions seem to be less damaging to *S. aureus in vitro*, and create a permissive environment for successful colonization (70). Human β-defensin-3 (hBD-3) is the peptide that seems to have the most potent anti-*S. aureus* effect *in vitro* and in skin infections (71–73). Its production from skin and nasal secretions is normally driven by the presence of *S. aureus* or by disruption of the skin barrier, implying it may have a role in clearance of *S. aureus* in both colonization and infection (74, 75). Its constitutive and induced levels in skin are significantly lower in persistent carriers as compared to non-carriers, and this pattern has recently been associated with *DEFB1* gene promoter polymorphisms (76, 77).

The multifunctional cutaneous cathelicidin LL-37 is impressively effective at *in vitro* killing of both extra- and intra-cellular *S. aureus* (78, 79). Despite this, in a single small study which included GPA patients, the nasal secretions of those colonized with *S. aureus* contained higher concentrations of LL-37 than non-colonized participants, and its production was induced by stimulation with *S. aureus* (75). The significance of this for the healthy population is unclear. The cathelicidin gene carries a vitamin D response element, and vitamin D increases expression and function of many AMPs (80, 81). An inverse association between vitamin D levels and *S. aureus* nasal carriage has been found in epidemiological studies (82, 83). However, vitamin D supplementation does not reduce persistent carriage in healthy adults (84). Host-derived lipids from sinuses and skin also exhibit anti-microbial properties (85–88). Several other peptides have been found to have anti-*S. aureus* activity but their role in colonization has not been assessed (89).

Some defects in local anti-microbial activity have been described in the skin of highly colonized populations. The skin surface of AD patients with particular filaggrin mutations is less acidic than healthy skin and exhibits inhibited AMP activity. *In vitro* experiments show increased *S. aureus* growth and expression of adherence and immune-evasion molecules under these conditions (90). Hexadecenoic acid and free sphingosine lipids are present at lower levels in skin of AD patients than healthy controls (91, 92). It is not clear if these skin defects are mirrored in the nasal epithelium or contribute to their higher nasal carriage rates. In GPA patients, aberrancies in baseline nasal mucosal cytokine expression and altered nasal epithelial AMP responses to *S. aureus* have been described but it is not known if this explains their increased colonization (75, 93).

The organism must overcome these local immune challenges if it is to persistently colonize. Unfortunately, *S. aureus* has adapted to this system by producing proteases to degrade AMPs, proteins to bind, and inactivate them, and by altering the charge of its cell wall to reduce their affinity to attach (94).

### The influence of host genetics on nasal colonization

In addition to the defensin gene polymorphisms mentioned above, several other mutations have been associated with nasal carriage. Interleukin-4 (IL-4), mannose-binding lectin, toll-like receptor 2 (TLR2), glucocorticoid receptor gene, and C-reactive protein polymorphisms have all been linked to carriage, as has HLA-DR3 (69, 95–97). The identified IL-4 polymorphism causes lower levels of IL-4, resulting in reduced mucin production and dampening of the Th2 response (98, 99). In a sample of elderly Dutch patients, the glucocorticoid receptor polymorphism found in persistent carriage was associated with a phenotype of putative high cortisol levels causing immunosuppression, and the non-carriage haplotype was thought to reflect reduced immunosuppression (100). Mechanistic explanations are not apparent for the other genetic traits to date.

Instead of interrogating for single genes, some studies have looked for individuals likely to share multiple genetic similarities to ascertain the role of host genome in colonization. Persistent carriage patterns were not concordant among same-sex siblings or even among twin pairs, regardless of gender or zygosity (101, 102). The fact that no strongly convincing genetic trait has accounted for successful or unsuccessful colonization indicates that it must be determined by multiple factors that may even differ from carrier to carrier.

## Expanding the “Host” Concept – Impact of the Microbiota on Host Immunity

Colonization with microorganisms begins during birth and continues throughout early life, such that each human is rapidly outnumbered by the diverse microbial community they carry. Where *S. aureus* is part of the nasal flora in particular individuals, it does not exist in isolation. The normal microbial population of humans is rapidly being characterized, and we share our upper airways with bacteria, fungi, and viruses – many of which are potentially pathogenic – during periods of both good and ill health (103–106). Some colonizing organisms at other sites may confer metabolic and/or immune benefits to their host, and dysbiosis may be associated with disease. Resident microbes have complex interplay, and can signal between species and across kingdoms, as well as directly modify host immune responses.

### “Interference” between *S. aureus* and neighboring resident microbes

The bacterial community of the nares in adults is variable, but dominated by *Corynebacterium, Priopionibacterium*, and *Staphylococcus* species (107, 108). The interaction between the host and each microbial species in the nares is also influenced by the other competing microorganisms present.

Traditionally, a resident *S. aureus* strain is thought to “hold fast” in its niche, whether by specific adherence factors or simply by physical occupation of space. It then resists later acquisition of different strains or even of the same strain (109, 110). This view has been somewhat challenged by more recent molecular methods showing that a minority of *S. aureus*-colonized individuals can carry more than one strain at a time or acquire new displacing *S. aureus* strains (22, 111, 112).

This “first-come-first-served” approach is also observed in its interplay with other staphylococcal species. *Staphylococcus epidermidis* colonizes almost 100% of humans, often with multiple strains concurrently (113). *S. aureus* carriage is negatively associated with *S. epidermidis* and *P. acnes* in adults (107). Resident *S. epidermidis* reduces but does not prevent *S. aureus* colonization in animal models following elimination of their original nasopharyngeal flora. This interplay may be due to genus-specific blocking of virulence gene expression, whereby *agr* auto-inducing peptides can act as inhibitors of quorum-sensing in a different staphylococcal strain or species (114, 115). Application of strains of *S. epidermidis* secreting the serine protease Esp inhibit *S. aureus* colonization *in vivo* and eliminate human nasal *S. aureus* carriage in pilot studies *in vitro* (116). This concept of inter- and intra-species bacterial interference is long-known, and appears most powerful between species of the same genus, as they often compete for the same ecological niche. In the 1960s, when a number of serious *S. aureus* epidemics occurred in hospital nurseries, it was noted that pre-existing colonization of the nasal mucosa or umbilical stump of infants prevented subsequent colonization by the epidemic strain. This observation led to the deliberate inoculation of neonates with a “low-virulence” *S. aureus* 502A strain that obviated colonization with the emerging penicillin-resistant strains and resulted in significant decreases in invasive *S. aureus* disease (117).

Other resident species in the nose behave quite differently. *Streptococcus pneumoniae* and *Haemophilus influenzae* strains acquired at different times can co-exist with the original species, and tend to have more transient periods of carriage (110, 118). Some studies show an inverse relationship between *S. aureus* and *S. pneumoniae*, but only in children and the association is not consistent (119–121). A mechanism has been proposed to explain this interspecies competition. Pneumococci produce sufficient hydrogen peroxide to induce a stress response and activate resident *S. aureus* lysogenic prophages. This results in staphylococcal cell lysis and death *in vitro* (122).

The nares have a temporally stable microbiota. Bacterial ecology is altered during the course of systemic antibiotic treatment, and intercurrent upper respiratory tract infections, but these alterations are short-lived (106, 123). Similarly, attempts to decolonize patients by using intranasal mupirocin and/or topical chlorhexidine is not a reliable strategy for long-term elimination, although it may decrease immediate risk of surgical site infections (124, 125). Repeated application of *Corynebacterium* species to the nares of persistent *S. aureus* carriers results in clearance for a variable period of time, and actively ingested probiotics fail to significantly alter *S. aureus* nasal carriage (126, 127).

Such “interference” is not confined to co-existing bacteria. Synergistic and antagonistic signaling may occur between kingdoms of normal flora. In murine infection models, co-infection with *Candida albicans* synergistically enhances virulence and mortality in systemic *S. aureus* infection (128). This is associated with an increase in pro-inflammatory cytokines and end-organ cellular infiltrates indicating fungal-bacterial modulation of host innate immune response (129). Conversely, the candidal quorum-sensing molecule farnesol inhibits *S. aureus* biofilm formation and compromises cell membrane integrity *in vitro* (130). Such modulatory cross-kingdom signaling is not well understood during asymptomatic *in vivo* colonization. Nevertheless, the prospect of altering the local constituents of the nasal microbiota to direct *S. aureus* colonization is tantalizing.

### The impact of the microbiota on immune development

The microbiota is overwhelmingly comprised of anaerobic bacteria residing in the distal gastrointestinal tract, although all mucosal surfaces are colonized with various microorganisms. Most knowledge of the interactions between colonizing organisms and host immunity relates to the intestinal microbiota. Colonization with these organisms provides benefits to the host by adding metabolic function and preventing pure pathogen overgrowth, but many gut microbes are also potentially pathogenic. Containing the growth of the vast number of “non-self” microbial cells in contact with the intestinal epithelium is a significant challenge to host immunity, but responding with over-zealous inflammatory activity results in host damage. Thus, while immune response must be present for health, it must also be tightly regulated and directed in a way appropriate to each tissue or organ site.

Induction and maintenance of immune tolerance to commensal intestinal organisms is essential to normal local and systemic lymphoid maturation (131–133). The microbiota orchestrates the differentiation and homeostasis of various T cell subsets in animals. It regulates development of pro-inflammatory intestinal Th17 cells, and anti-inflammatory regulatory T cells (Tregs) both in the intestine and systemically (134). Tregs are greatly reduced in germ-free animals, and this depletion results in detrimental inflammation due to expansion of unopposed microbe-specific pro-inflammatory T helper subsets (135, 136). Conversely, in skin, commensals normally drive pro-inflammatory tissue-resident T cells preferentially, and germ-free animals have greatly increased numbers of skin Tregs (137).

Much less is known about the mechanisms and outcomes of microbial immunomodulation in humans, but our host immune responses may also be manipulated to favor a colonizer’s persistence. Co-culture of human peripheral blood mononuclear cells (PBMCs) with species of *Lactobacillus* and *Bifidobacterium* show differences in the induction of Tregs specific to those species (138). *C. albicans* produces prostaglandins that reduce lymphocyte proliferation, TNF-α and chemokine production while upregulating IL-10 production in mammalian cells (139). Such immunomodulation of effector and Treg response mechanisms by intestinal microbes has also been implicated in protecting against development of systemic allergic and autoimmune disorders (140–142).

### The imprint of the microbiota on human adaptive immunity

In animals with controlled mucosal exposure to gut commensal antigens, the development of specific immune responses is limited to the mucosal and local mesenteric lymphoid organs without spread to secondary lymphoid organs or development of systemic immunity (143). T lymphocytes are essential to this compartmentalized response and their depletion results in systemic microbial translocation (144).

While in a tolerant co-existence with the microbiota, humans nonetheless often develop a systemic adaptive immune response to these organisms, perhaps as a result of transient bacteremias due to such microbial translocations. Circulating antibodies to commensal microbial antigens including *C. albicans, Escherichia coli, Clostridium difficile, Neisseria*, and *Bacteroides* species are common in healthy individuals (145–149). These antibody responses are significantly elevated in some cases of chronic or acute intestinal barrier disruption (150–152). Adaptive cellular responses are also normally produced, with small numbers of *E. coli*-specific Th1 cells present in the peripheral blood of healthy individuals (153). Experimental gastroenteritis and subsequent translocation of intestinal bacteria enhances systemic microbiota-specific memory Th1 cell development (154). Despite the presence of these primed B and T lymphocytes, they are not associated with ongoing uncontrolled systemic inflammatory responses in the absence of invasive infection. Instead, the compartmentalized mucosal immune response tolerates but tightly confines the intestinal microbiota to its appropriate site. Much less is known, however, about the impact of the microbiota at other sites on local and systemic immune response.

### Site-specific adaptive immunity

The establishment and maintenance of balanced interactions between the host and its microbiota seem a key requirement for health, but little is known about the unique immunomodulation by most pathobionts in humans. Understanding these mechanisms and translating the findings into therapeutic interventions remains a major challenge but an attractive avenue for future vaccine development. Both pro- and anti-inflammatory antigen-specific lymphocytes may be induced by bacterial colonization of extra-intestinal sites as well. The polarization and efficacy of these cells may in fact be completely dependent on the site or compartment at which they first encounter the immune system. In an animal model of *Listeria monocytogenes* infection, for instance, intravenous inoculation drives the development of systemic long-lived Th1 effector memory cells, whereas intranasal infection with the same organism drives short-lived central memory Th17 cells (155).

In humans, this concept of site-specific adaptive immunity has been elegantly explored in the case of *S. pneumoniae*. Various pneumococcal-specific T cells in humans – Th1, Th17, Tregs – are much more numerous in tonsillar lymphoid tissue close to the site of colonization, than in the peripheral blood (156). Similar mucosal Th17 responses to experimental colonization are seen in mice and humans, and this protects against subsequent colonization in both (157, 158). Human tonsillar lymphocytes produce pro-inflammatory IL-17A in response to pneumococcal antigens, which improves *in vitro* phagocytic killing of the organism (159). In contrast, adenoidal tissue of children naturally colonized with *S. pneumoniae* shows increased proportions of IL-10-secreting pneumococcal-specific Tregs, which inhibit CD4^+^ proliferation and production of pro-inflammatory cytokines (IFNγ, TNF-α, and IL-17A) (160). The relative proportions of pro- and anti-inflammatory T cells specific to *S. pneumoniae* in tonsillar lymphocytes are roughly equal. These local immunomodulatory Tregs may thus reduce inflammation-related airway damage during infection, but facilitate the persistence of pneumococcal carriage.

In peripheral blood lymphocytes, the picture is very different. The circulating immunosuppressive Treg phenotype is far less evident and the balance is overwhelmingly skewed in favor of Th1 and/or Th17 cells (156, 159, 161). This compartment is primed toward a rapid pro-inflammatory cellular response, and also has ready circulation of anti-pneumococcal antibodies, both of which are critical for efficient bacterial clearance in invasive disease. This shows that the bias of appropriate host response is site-specific, and anti-pneumococcal cells’ function and mechanisms of protective immunity may also vary by site. Normal host response is tailored to a balanced tolerance at sites normally colonized by commensal organisms, and rapid attack at normally sterile sites. This tightly regulated balance between pro- and anti-inflammatory responses to *S. pneumoniae* seems to greatly influence the outcome of colonization and perhaps even that of infection (157, 162).

Such site-specific characterization of local and systemic immune response to colonization, and exploration of the relative importance of these in the prevention of invasive disease have only been minimally elucidated for *S. aureus* to date and are further discussed below.

## The Effect of *S. aureus* Colonization on the Host Immune System

In the battle between microbe and host during infection, many *S. aureus* attributes that contribute to its virulence and lethality have been described, but much less is known about the host’s defense or breaching of this defense. Where this has been studied it is usually in the context of invasive disease, hence there is extremely limited knowledge of host response during asymptomatic periods of colonization. Critical questions remain unknown. Are there particular protective actions preventing colonization? What host immune failures occur to allow invasion? And does the immune imprint of colonization affect subsequent response to invasive infection?

### The influence of colonization on outcome of infection – a hint that colonization matters?

Colonization is known to substantially increase the risk of subsequent infection, and invasive *S. aureus* disease carries a high mortality rate. On the other hand, it is clear that being a carrier alone – like a large proportion of the healthy population – does not cause death or other adverse consequences in the absence of infection. It is unknown whether host immunological adaptation to the colonizing strain in nasal carriers confers any advantage or disadvantage in recovery from active infection.

One notable large-scale Dutch study retrospectively examined the incidence of nosocomial *S. aureus* bacteremia and mortality in carriers (persistent and/or transient) vs. non-carriers in a 120-day follow-up period (37). As expected it showed a higher incidence of bacteremia among carriers, although all-cause or infection-attributable mortality was not significantly different between both groups (0.1 vs. 0.1%; *p* = 0.81 for *S. aureus*-attributable deaths). However, when the subset who did develop nosocomial bacteremia were analyzed independently, carriers appeared to have a lower all-cause and *S. aureus*-attributable mortality (18 vs. 47%; *p* = 0.005 and 8 vs. 32%; *p* = 0.006). Severity of disease and incidence of septic shock were not reported. The carrier group were significantly younger and had fewer cardiac issues, which may explain their more favorable outcomes. Intriguingly, however, there may have been a key difference in their immune responses. The carriers were a more “immunocompromised” group (35 vs. 12%; *p* = 0.02) – although this is unfortunately not further defined – which may have globally dampened potentially harmful “over-zealous” innate and adaptive responses in the setting of sepsis. Alternatively, they may have had a more appropriate or well-orchestrated specific response to invasive disease, given their prior exposure and a degree of potential immune tolerance to *S. aureus*.

A meta-analysis looking at the few observational studies that have examined the association between pre-morbid *S. aureus* colonization and mortality showed that carrier status showed a similar non-significant trend in reducing mortality directly attributable to the infection (163).

The immune mechanisms induced by colonization that might result in such improved outcomes following infection are not defined, although some clues exist. In humans, high titers of anti-TSST-1 seem to be protective against staphylococcal toxic shock syndrome in that the disease seems to occur in those without protective anti-TSST antibodies (164). Persistent nasal carriers have higher titers of neutralizing antibodies to several superantigens (sAgs) that significantly reduce T cell proliferation and activation (165). This may lower their risk of developing toxic shock syndrome or attenuate the severity of sepsis. Higher levels of antibodies against several *S. aureus* toxins just prior to or at the onset of infection decreases the likelihood of developing sepsis during bacteremia, and although pre-morbid colonization was not formally assessed, the patients with improved outcome had a history of *S. aureus* infections (166). Establishing whether there is any association between the immune imprint of *S. aureus* colonization and the mortality attributable to this infection is critical for orchestrating and predicting response to infection and vaccines in future patients.

### Humoral response to *S. aureus* colonization

Recognition and handling of *S. aureus* by the innate immune system is notable and has been outlined elsewhere (167–169). This type of immunity is currently considered to lack specific memory, and thus is not as attractive a target as the adaptive immune system for vaccine research (170). Humoral immunity is more enticing and established, but there a number of caveats against its promise in the case of *S. aureus*. B cell deficiencies in humans are not associated with increased infection rates, and do not worsen outcomes in animal challenge models (171–173). *S. aureus* is uniquely armed with protein A to eliminate antibodies formed against it by binding to their Fcγ domain and by interacting with B cells to ultimately cause their anergy and apoptosis (174–176). This leads to a compromised adaptive immune response against other *S. aureus* antigens. Serum antibodies certainly seem to have functional antibacterial behaviors *in vitro*, but there may well be other staphylococcal products inhibiting optimum antibody activity *in vivo* (177, 178).

Frequent exposure to *S. aureus* does indeed imprint a memory antibody response in the host, although to a varying extent. The mechanism of induction of antibodies by colonization is not established. Transient bacteremias, self-resolving minor infections or absorption of toxins across the mucosa could directly explain systemic immune exposure to microbial antigens (179). Colonization alone less easily explains the production of adaptive immune memory. It only results in antibody formation to a limited selection of the *S. aureus* antigens known to be present, and experimental nasal colonization in humans does not induce significant humoral changes (180, 181).

Whether antibodies are implicated in preventing colonization – by inhibiting adherence, facilitating immediate clearance, or other unknown methods – is also undetermined. It is clear that transplacental transfer of a lifetime’s collection of maternal anti-*S. aureus* IgG does not protect infants from colonization in infancy, nor does the development of their own anti-*S. aureus* antibodies prevent subsequent colonization (182, 183). Most adults and children have a variable degree of anti-*S. aureus* antibodies of various classes present in serum, whether colonized or not (178, 183, 184). Local antibodies in the nares are less studied although show some correlation with systemic titers (178).

Different studies have found lower or higher levels of antibodies against *S. aureus* antigens among nasal carriers and non-carriers (64, 177, 178, 182, 184). The overwhelming trend is of considerable inter-individual variation, and findings are often contradictory. Consistently reproducible key patterns of antibody titers or differences in functionality between carriers and non-carriers have not been shown. Unfortunately, not all studies have rigorously identified true persistent carriers before drawing conclusions about differences in antibody levels, and those that have may be more reliable (11, 178, 180).

Some animal studies have shown antibody-based interventions to prevent *S. aureus* colonization. Production of antibodies to IsdA or IsdH prevented nasal colonization of cotton rats, but only when a lower bacterial inoculum was used (64). Intranasal immunization of mice with recombinant ClfB or systemic administration of anti-ClfB monoclonal antibody reduced bacterial load but did not prevent colonization (185). Immunization to prevent colonization in humans has not been tested.

Whether in infection or colonization, antibody patterns are extremely diverse and it is difficult to discern clear patterns. Of course, only a fraction of the antigens in *S. aureus*’s protein and polysaccharide repertoire have been evaluated for antibody response thus far, and perhaps combining patterns of multiple antibodies may better discriminate between groups. Nonetheless, even patients infected or colonized with genetically similar organisms produce unique responses (186). This further supports the theory of a uniquely personalized host-microbe relationship dependent on the temporal history of exposure, number, and genetic diversity of strains and intrinsic adaptive host response.

### Adaptive cellular immune response to *S. aureus* colonization

Evidently, colonization – or perhaps more accurately transient microinvasions or other exposures to *S. aureus* – influences systemic antibody repertoire. It is equally likely that this history of exposure induces adaptive cellular immune responses, and of course T helper cells are essential for optimal B cell activity. There is extremely limited data on this in the case of *S. aureus* colonization.

Early intestinal colonization with *S. aureus* in children is associated with increased numbers of systemic IL-4 and -10 producing cells (187). It may also predict a higher likelihood of atopic disease (188). Airway exposure to *S. aureus* enterotoxins has been linked to the development of asthma and allergic rhinitis, perhaps by inducing local Th1/Th17 responses (189–191).

**Figure 2:**
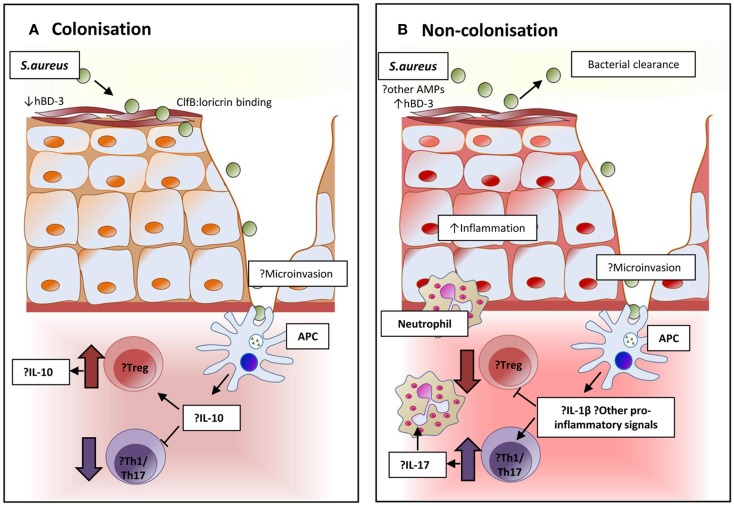
**Proposed immunomodulation affecting and resulting from colonization in persistent and non-persistent carriage**. **(A)** The epithelium and local environment of the nares in individuals with *S. aureus* carriage may be more favorable for colonization with high levels of host ligands to facilitate adhesion, and reduced concentrations of the potent anti-staphylococcal peptide human β-defensin 3 (hBD-3) in nasal secretions. Interaction and processing of *S. aureus* by local antigen-presenting cells may result in an immune tolerance and suppression of pro-inflammatory responses. Inhibition of bacterial clearance would allow persistent colonization. **(B)** In non-carriers, the local environment and response might resist successful *S. aureus* colonization. Nasal secretions may contain higher levels of hBD-3 or other anti-microbial peptides. Local immune response to the organism could be more pro-inflammatory and promote the expansion of Th17 cells to attract neutrophils and create local inflammation that facilitates bacterial clearance.

T cells in nasal lymphoid tissue mediate clearance of *S. aureus* from nasally inoculated mice. This decolonization is dependent on the Th17 response and facilitated via IL-17A and its associated neutrophil influx (192). Unlike humans, mice are naturally somewhat resistant to nasal colonization, and further understanding of these Th17 responses could be used to develop interventions to reduce or understand human colonization. The mechanisms of local and systemic cellular responses to nasal colonization and their relative importance in the prevention of invasive disease have not been fully elucidated for *S. aureus* to date, although some evidence points to suppression of local pro-inflammatory signals facilitating persistent colonization (193–195). A proposed mechanism for nasal carriage and resistance to carriage is presented in Figure 2.

## The Importance of T Cells in *S. aureus* Infection and Their Modulation of Immunity

Some animal and preliminary human data has proposed an important role for various T cells in dealing with certain *S. aureus* infections. However, as discussed above, human exposure to the organism long prior to an episode of infection will likely determine the nature of the T cell response re-activated during later disease. Understanding how T cells are “primed” during colonization is fundamental to manipulating their activity for adjunctive treatment or vaccination.

### The role of T cells in animal models of *S. aureus* infection

In murine wound infection models, T cell deficient mice exhibit lower concentrations of CXC chemokines in local tissue due to a reduction in lymphocyte-derived IFN-γ (196). This results in less trafficking of CD4^+^ cells and neutrophils to the site, reduced inflammation, and lower bacterial burden (197). Absence of gamma-delta T cells producing IL-17A also leads to a similar phenotype (198, 199).

In systemic infection T cells appear to be crucial to survival with rapid mortality following intravenous challenge in T cell knockout mice (173). Th1 and Th17 subsets seem most critical, and both IFN-γ and IL-17 are routinely produced during systemic infection (200). Deficiency in IFN-γ but not IL-17A results in increased mortality (201, 202). However, Th17 expansion may be essential to effective vaccine responses (202, 203).

### The role of T cells in human *S. aureus* infection

With the exception of the superantigens, few *S. aureus*-specific T cell epitopes have been identified, and the normal human T cell response to this organism has had negligible evaluation (204–207). There have been no human studies examining the role of T helper cells in *S. aureus* colonization or invasive infection, but there are some clues as to the importance of T cells in human *S. aureus* disease.

Particular T cell subsets have been implicated in intact cutaneous and mucosal immunity to *S. aureus*. A heterogeneous group of disorders may cause the chronic mucocutaneous candidiasis (CMC) syndrome, which is characterized by integumentary T cell hypo-responsiveness to *C. albicans* antigens (208). These patients suffer from protracted *C. albicans* and *S. aureus* mucocutaneous infections. Inborn errors of Th17 cells seem to underlie CMC (209, 210). Patients with the rare autosomal dominant hyper-IgE syndrome (AD-HIES) are prone to CMC, and to staphylococcal skin and lung abscesses along with other abnormalities (211, 212). Mutations in the signal transducer and activator of transcription 3 (STAT3) gene account for the majority of cases (212). STAT3 is involved in the signal transduction of several pro- and anti-inflammatory cytokines, and stimulation of PBMCs from these patients shows increased secretion of TNF-α and IFN-γ (213). Naïve CD4^+^ T cells with this mutation fail to differentiate into Th17 cells, but retain the ability to differentiate into other subsets (214, 215). Interestingly these patients do not seem more prone to *S. aureus* bacteremias despite their global systemic Th17 deficiency. This may be explained by differences in site-specific immune response. Unlike cells from other sites, human skin and respiratory epithelial cells seem to require the synergistic stimuli of both Th17-derived and classical pro-inflammatory cytokines for enhanced production of AMPs (h-βD-2 and -3) and neutrophil chemotaxins (216).

HIV-infected individuals are also commonly affected with recurrent skin and mucosal infections, frequently caused by *C. albicans* and *S. aureus*. Of all CD4^+^ cells, HIV-positive patients preferentially show profound loss of circulating Th17 cells, even at early disease stages (217, 218).

Th17 cells thus seem essential for intact mucocutaneous immunity to extracellular bacteria and fungi. Specific T cell responses in infections of other sites are less well understood.

### T cell immunomodulation induced by *S. aureus* and its effects

The multiplicity of mechanisms utilized by *S. aureus* to evade the innate immune system and cause infection are staggering (219). Its means of skirting the adaptive immune system are less well appreciated but its ability to delete or block development of evidence of its presence must surely contribute to the lack of an effective immunological memory. Invasive *S. aureus* infections are associated with decreased transcription of genes relating to adaptive immunity and increased expression of myeloid and innate immunity genes (220, 221). Several microbial factors are known to directly interact with lymphocytes (Table 1).

**Table 1:** **Staphylococcal factors implicated in directly modulating the host adaptive immune response**.

Immunomodulatory factor	Prevalence in clinical strains (%)	Evidence for activity in colonization	Human target	Effect
Protein A (Spa)	91 (222)	Mostly transcribed in persistent carriers (58, 222)	(i) Fc region free IgG; (ii) B cell-surface IgM V_H_3 region	(i) Inhibits opsonophagocytosis; (ii) programed B cell death
Superantigens (staphylococcal enterotoxins and toxic shock syndrome toxins)	73 (223)	Variably transcribed during carriage (181)	MHC Class II	Binds MHC Class II to the T cell receptor to cause initial activation followed by anergic unresponsiveness
MHC Class II analog protein (Map)	94 (222)	Unknown	MHC peptide-binding groove	(i) Reduced lymphocyte proliferation; (ii) Th2-predominant response
Leukotoxin ED	30–87 (224, 225)	Unknown	CCR5 (T lymphocytes and macrophages)	Cell membrane pore formation causing cytotoxicity

Secreted or wall-anchored staphylococcal protein A binds IgG in the incorrect orientation for neutrophil recognition and thus inhibits opsonophagocytosis. It also binds to the V_H_3 region of IgM on the surface of B lymphocytes, initiating a B cell receptor-mediated programed cell death that results in significant depletion of the reservoir of potential antibody-producing cells (174). The presence of an array of superantigenic toxins directly activates vast numbers of T cells, but this is followed by the loss of these cells’ ability to respond to these antigens (anergy). This prevents normal development – via MHC Class II/TCR presentation of processed antigens – of true microbial antigen-specific effector and memory cells (226). At least one of these superantigens is expressed by most circulating clinical isolates (223, 227). The MHC Class II analog protein (Map) is a secreted *S. aureus* protein with similarity to the MHC peptide-binding groove. Its binding reduces lymphocyte proliferation and shifts this response in a Th2 direction, thus suppressing the Th1 responses shown to be important for bacterial clearance in animal systemic infection (228). CCR5 on the surface of macrophages and T lymphocytes appears to be necessary for Leukotoxin ED (LukED) to produce pore-forming cytotoxicity, and absence of either host CCR5 or staphylococcal LukED results in lower levels of pro-inflammatory cytokines and markedly reduced mortality in systemic infection models (229). However, these factors have largely been shown in animal infection models or *in vitro*, without necessarily simulating physiological concentrations. More importantly, none of them have been exclusively examined for their immunomodulatory effects in the setting of colonization.

The balance between pro-inflammatory and anti-inflammatory adaptive responses required for controlled but successful bacterial clearance and clinical recovery are unknown, as is whether or not colonization influences this balance. Repeated or prolonged *S. aureus* encounters may lead to an altered and even immunosuppressive response to its antigens at certain sites.

Children with AD and *S. aureus* skin colonization have globally reduced IFN-γ production from CD4^+^ PBMCs in response to non-specific stimulation, particularly those with higher bacterial loads (193). This finding may, of course, be more to do with the underlying disease than the organism. Human monocyte-derived IL-10 has been experimentally shown to reduce development of pro-inflammatory Th1/Th17 responses *in vitro* (230). This inhibitory response is far less dramatic when dendritic cells are the antigen-presenting cells (APCs), which may support the site-specific concept of aggressively responding to invasion of normally sterile sites like the bloodstream. Using monocytes to prime naïve human T cells with heat-killed *S. aureus* produces antigen-specific populations of Th1 and Th17 “pro-inflammatory” cells. However, persistent restimulation of these *S. aureus*-specific Th17 cells results in a switch to an “anti-inflammatory” phenotype – reduced IL-17 and increased IL-10 production. The same pattern was seen in *S. aureus*-specific human memory Th17 and Th1/Th17 cells (231). These immunomodulatory responses may occur upstream of the adaptive response, as interactions of the innate immune system with *S. aureus* produce cytokines to polarize T cell production and memory responses. For instance, staphylococcal peptidoglycan uses TLR2 signaling to induce IL-10 production and apoptosis of human APCs (232).

Similar immune “switching” is seen in animals after exposure, where these effects can then be evaluated in infection challenge experiments. Persistent systemic exposure to *S. aureus* in mice also leads to *in vivo* T cell clonal anergy and immunosuppression which may be IL-10 mediated (233–236). Intraperitoneal vaccination of naïve mice with heat-killed *S. aureus* switches their cytokine pattern on subsequent intravenous challenge. They show decreased IL-17, unchanged IFN-γ, and increased IL-10 production, as compared with unvaccinated mice (200). Staphylococcal peptidoglycan exposure increases plasma IL-10 and reduces IFN-γ and TNF-α concentrations in response to intravenous bacterial challenge even in the absence of intact TLR2 signaling. Such “primed” immune-tolerant mice repeatedly demonstrate improved bacterial clearance and significantly reduced mortality in systemic *S. aureus* disease (98, 232, 237).

It could be proposed that inducing a degree of *S. aureus*-specific immunosuppression may be a useful defensive adaptation against the pro-inflammatory “cytokine storm” that would be expected if superantigen-provoked massive T cell activation was allowed to go unopposed. It is remarkable that despite the prevalence and transcription of multiple superantigens in clinical infections, toxic shock syndrome is much more rarely observed than *S. aureus* bacteremia (223). Staphylococcal superantigen stimulation induces both TCR-mediated clonal anergy and Tregs producing IL-10 (235, 238). Rather than being protective, however, high levels of serum IL-10 at presentation in *S. aureus* bacteremia patients strongly predicts their mortality, although this is a relatively crude measurement and its cellular source or specificity in this setting is not determined (239).

Successful colonization in mice seems to be facilitated by an immunosuppressive predominance, and clearance dependent on developing specific pro-inflammatory (Th17) responses. Colonization in humans may mirror this pattern. The effects colonization has on innate immune signaling, polarization of systemic adaptive immunity, and whether these influence clinical outcome during subsequent infections is completely unknown. Closing these knowledge gaps is essential to developing an effective vaccine.

## Anti-Staphylococcal Vaccine Design is Complicated by Colonization

An effective vaccine to prevent *S. aureus* disease remains elusive. Mathematical models conclude a vaccine of even relatively limited efficacy (≤10%) would significantly decrease the incidence of invasive disease and be extremely cost effective in high-risk populations (240–242). Some important points on colonization’s impact on immunity and the challenges of producing such a vaccine are illustrated in the most important human studies to date.

### Consideration of colonization in important human anti-*S. aureus* vaccine studies to date

Passive and active immunization strategies have been evaluated to prevent occurrence of or improve outcome of *S. aureus* bacteremia (243–249). Only two anti-*S. aureus* vaccines have progressed to disappointing Phase III clinical trials and those that have completed Phase II have not suggested signs of efficacy to date. All have aimed to use antibodies to mediate their effect. Production and adoptive transfer of these antibodies showed promising protection in animal challenge models in all preclinical studies. The largest clinical studies raise the possibility that colonized individuals may respond differently to vaccination.

#### CP5 and 8 immunization

The most prevalent *S. aureus* capsular types 5 and 8 (CP5 and 8) were conjugated to a protein carrier and this vaccine was tested in a hemodialysis population (243). Despite high antibody titers in vaccinees, a reduction in *S. aureus* bacteremia was not observed, although not all clinical isolates expressed CP. Twenty-two percentage of the participants were colonized prior to vaccination where colonization was defined as two out of two positive cultures for *S. aureus* from swabs of the anterior nares 2 weeks apart. Tunnel exit site colonization was not reported. They observed that while nasal carriage was a risk factor for increased bacteremia (7.6 vs. 3.1 per 100-person-years; *p* < 0.01) among placebo recipients as expected, in vaccine recipients, the rate of *S. aureus* bacteremia among carriers was not higher than that of the non-carriers (3.0 vs. 3.1 per 100-person-years; *p* = 0.82). Although the numbers who developed invasive disease were small, it is an interesting observation suggesting that nasal carriage may induce a different response to vaccination.

A smaller study of the same vaccine in healthy volunteers looked specifically at its impact on colonization (250). All had comparable antibody response post-vaccination, regardless of prior colonization status, but nasal colonization rates were not significantly affected.

#### IsdB immunization

In a murine model, immunization with the highly conserved iron-scavenging protein IsdB showed decreased mortality in a subsequent intravenous challenge with live *S. aureus* and seemed to correlate with anti-IsdB antibody titers (251). It was also shown to confer T cell-mediated protection by expanding a Th17 antigen-specific population, and adoptive transfer of these antigen-specific CD4^+^ cells conferred protection (203). This dual activation of humoral and cellular immunity in animal models, as well as the antigen’s specificity for and conservation across *S. aureus* strains, made IsdB a highly attractive molecule for vaccination. Healthy humans have baseline anti-IsdB antibodies – presumably due to prior exposure and colonization – and titers have been shown to increase significantly between acute and convalescent samples in patients with *S. aureus* bacteremia as compared to patients with alternative infections, suggesting this response may play a role in recovery (252).

Phase IIa studies were carried out in chronic hemodialysis patients, and produced increased antibody responses in vaccinees, and increased opsonophagocytic functional activity in a subset (253). Despite the fact that 35–84% of these patients tend to be colonized with *S. aureus*, carriage rate was not reported, although prior exposure was suggested as an explanation for the brisk response and higher antibody titers seen following vaccination as compared to healthy volunteers (12). In Phase III pre-cardiothoracic surgery patients, vaccination produced increased anti-IsdB titers, but failed to significantly reduce the incidence of *S. aureus* bacteremia, deep sternal wound infections, or all-cause mortality (249). However, the trial was terminated early due to increased mortality among vaccine recipients who developed *S. aureus* infection vs. placebo recipients who did, especially among those with MRSA disease. Nasal colonization rates were similar between vaccine and placebo groups, although criteria used to define this were not shown. As would be expected, invasive disease was higher among carriers than non-carriers, although these groups seemed to respond differently, just as with the CP5 and 8 vaccine trial. Vaccine efficacy appeared slightly greater in the vaccinated carrier group in *post hoc* analysis – invasive disease incidence was 3.3% in vaccinated carriers vs. 5.5% in placebo carriers (*p* = 0.09).

### Defining the purpose of an anti-*S. aureus* vaccine

**Figure 3:**
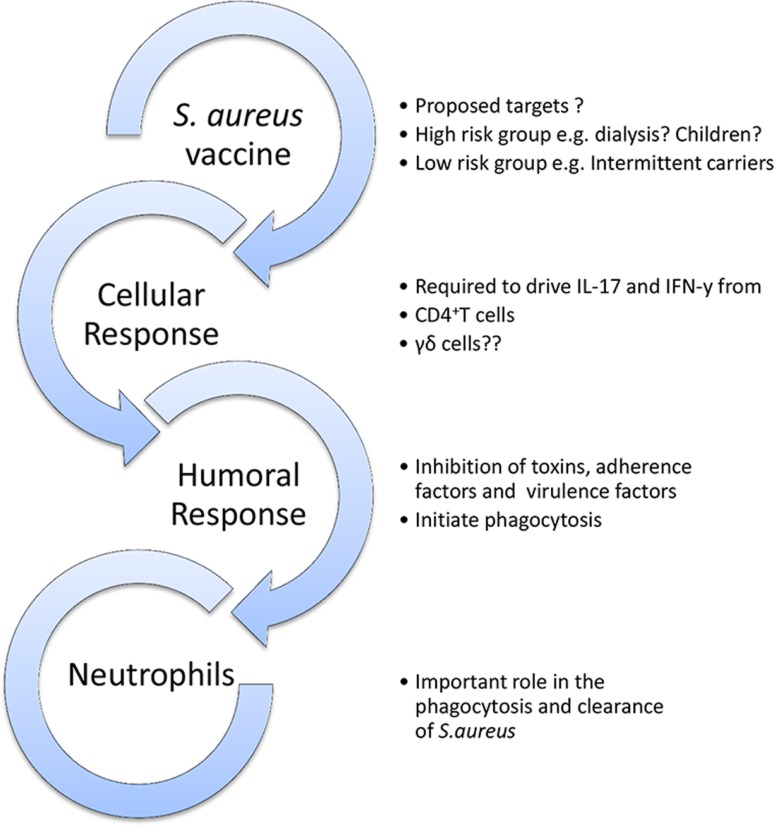
**Outline model of a protective vaccine against *S. aureus* infection**. A vaccine may be universal or specifically target high-risk groups. It should ideally aim to elicit humoral, cellular, and phagocytic responses.

A candidate vaccine must have a clearly defined purpose (Figure 3). Should a vaccine aim to prevent colonization? To prevent invasion? To augment the immune response to invasion and thus attenuate severity of disease? Should it target high-risk patient groups or be universal? Should children or infants be targeted for vaccination as their higher carriage rates make them greater potential reservoirs for transmission? What antigens, route of delivery, or adjuvant should be used? What collateral effects can be expected? Could there be a herd immunity effect? Might there be unexpected and perhaps harmful alterations in the balance of the local microbiota?

### The potential impact of *S. aureus* vaccines on nasal colonization

Unlike *S. aureus*, many other nasopharyngeal pathobionts have well-characterized disease-causing serotypes, or they mediate their disease by secreting local toxins rather than systemic invasion. Several also have known mechanisms of bacterial clearance (e.g., opsonizing antibodies to facilitate phagocytosis) and an established protective immunity effect whereby natural infection prevents against repeat episodes of disease. For *S. aureus*, none of these is known. As such, the development of a vaccine is significantly more challenging.

Since the introduction of widespread childhood vaccination, nasopharyngeal colonization rates have dropped for *Corynebacterium diphtheriae, H. influenzae* capsular type b (Hib), and vaccine serotypes of *S. pneumoniae* (254–256). This is in spite of the fact that many of their vaccines have specifically “anti-disease” effects and may not have been expected to influence carriage, other than reducing the efficient bacterial spread by those with disease.

The status of *S. aureus* as a harmless commensal organism in a significant proportion of the population complicates vaccine design. Colonization is the greatest risk factor for disease, yet the vast majority of carriers will never develop invasive disease. Importantly, no evolutionary or other advantage has been conclusively observed among persistent carriers of *S. aureus*, and conversely, no convincing disadvantage observed among those who seem resistant to colonization. Defining the differences between carrier and non-carrier groups has proved difficult. They are most likely multifactorial – differences in host genetics and epithelial cell molecules, differences in co-existing microbiome, and perhaps differences in *S. aureus* that make some strains superior colonizers. Difficulties in establishing clear differences between colonizing and disease-causing strains would suggest that vaccine candidates effective at producing protection against *S. aureus* infection may also protect against colonization as an unintended collateral effect. If so, absence of *S. aureus* may alter the normal balance of the nasal microecology with potentially unpredictable results. Overgrowth of competing pathobionts could result in their causing invasive disease, or as yet unknown favorable effects of *S. aureus* on local and systemic immunity could be lost.

### Considering more than antibody response in vaccine design

Inducing humoral immunity would seem the most logical place to start, given its success in vaccines against *S. pneumoniae, N. meningitidis*, and *H. influenzae*. In comparison to adaptive cellular responses, antibody responses are easier to measure, and depend only on the conserved tertiary structure of a protein or polysaccharide, rather than the unknown peptide conformation of an antigen after HLA-restricted processing and presentation by APCs. However, despite multiple target antigens successfully inducing antibodies, this approach alone has not been successful. This is perhaps not surprising in light of mounting evidence that *S. aureus* has a significant propensity to “hide” intracellularly, and antibodies target extracellular pathogens (257). Additionally, the presence of staphylococcal protein A on the cell wall is a crafty anti-humoral defense mechanism, enabling the organism to immobilize antibodies on its surface and thus render them ineffective. Most importantly, clinical evidence that antibodies are important mediators of protective immunity is completely lacking, whereas cellular immune response seems to have a role in determining response to infection and perhaps colonization. It may also be likely that specific cell subsets are involved in protection against disease only in certain sites – for instance, Th17 cells appear relevant in skin and mucosal disease but are not convincingly important once systemic invasion has occurred (199, 202, 258). As such, numerous mechanisms to ensure multi-site immunity should be employed. It is now widely accepted that anti-*S. aureus* vaccine strategies may need to drive both humoral and cellular immune responses to confer efficient protection, and will probably require multivalent antigens and perhaps a prime-boost approach.

### The lack of an analogous animal model for challenge studies is problematic

A concerning issue is the use of experimental animals who are not natural hosts for *S. aureus* in research for future vaccines. Studying candidate vaccines in rodent models has inherent limitations due to well-known differences in anatomy and immunobiology (259). Vaccine strategies shown to induce both cellular and humoral immune responses in rodents may not protect even these animals in challenge studies (260). There currently is no model truly analogous to humans to test the influence of natural and dynamic colonization on potential vaccines (261, 262).

Many animal studies define the efficacy of their interventions as a reduction in quantitative bacteremia or organ bacterial load, rather than sterile protection against infection. In humans, however, a threshold of “tolerable” or “safe” *S. aureus* bacteremia has not been found. Even endovascular infections have bacterial loads <1000 CFU/mL in humans, and the reduction in organ bacterial loads reported as success in vaccinated animal studies may be completely irrelevant for human infections (263–265). Rather, the presence of *S. aureus* in a blood culture at any level is always considered clinically significant (266). Perhaps unsurprisingly, many interventions shown to reduce staphylococcal sepsis in animal models of systemic disease have repeatedly failed to translate into a clinical effect in humans.

Regardless of their colonization status at the time of enrollment in a clinical study – unlike their laboratory animal counterparts – human participants are certainly not immunologically naïve to *S. aureus*. Initial exposure may prime the immune system and alter its response to subsequent bacterial encounters. Repeat antigen exposure may polarize and drive different cellular responses in humans than those we may hope for or expect from animal models. No candidate vaccines to date have been tested in challenge models in species that can be naturally colonized with *S. aureus*. A lifetime’s exposure to *S. aureus* may leave a critical imprint on a person’s immunological memory that affects subsequent response to vaccination.

## Conclusion

The human immune system readily recognizes and mounts specific responses to *S. aureus* antigens in settings of transient exposures, persistent colonization, local, and systemic disease. Immunogenicity does not appear to be the problem, but producing a lasting protective immunity remains elusive. The major problem facing vaccine researchers is that correlates of immune protection in human *S. aureus* colonization and disease are not sufficiently understood, and whether a protective immune response can in fact be produced in humans is unknown. Thus, choosing markers to measure the efficacy of vaccines in preclinical studies is extremely challenging. It seems that gaining a true understanding of host-pathogen interactions, both in health and disease, should be an immediate focus of research.

Colonization is the greatest risk factor for infection, and may modify its outcome. As such, it is essential that its impact and relevance should be routinely assessed in future clinical studies. Exposure to *S. aureus* through colonization may have immunomodulatory effects on the cellular and/or humoral responses that could potentially influence vaccine-induced immunity, and thus different populations may produce different responses. Outcomes measured should routinely extend beyond antibody titers – which are of questionable significance – and include changes in the frequencies or phenotypic response of specific T lymphocyte populations.

It seems foolhardy to ignore the immunological memory created by colonization in trials that aim to assess the immunological effects of candidate anti*-S. aureus* vaccines in specific populations. It is completely unknown whether suppression or enhancement of particular cellular responses during human colonization and disease has any effect on the prevention or clearance of invasive infection. Until we understand how nasal colonization impacts host immune response, we will continue to immunize in the dark.

## Author Contributions

Aisling F. Brown and John M. Leech drafted the manuscript. Thomas R. Rogers reviewed the manuscript. Rachel M. McLoughlin conceived the outline and helped draft the manuscript.

## Conflict of Interest Statement

The authors declare that the research was conducted in the absence of any commercial or financial relationships that could be construed as a potential conflict of interest.
